# Prevalence and Relatedness of *mcr-1*-Mediated Colistin-Resistant *Escherichia coli* Isolated From Livestock and Farmers in Japan

**DOI:** 10.3389/fmicb.2021.664931

**Published:** 2021-04-26

**Authors:** Akiyo Nakano, Ryuichi Nakano, Ryuji Nishisouzu, Yuki Suzuki, Saori Horiuchi, Takane Kikuchi-Ueda, Tsuneyuki Ubagai, Yasuo Ono, Hisakazu Yano

**Affiliations:** ^1^Department of Microbiology and Infectious Diseases, Nara Medical University, Nara, Japan; ^2^Livestock Food Agriculture Course, Soo High School Kagoshima, Kagoshima, Japan; ^3^Department of Microbiology and Immunology, Teikyo University School of Medicine, Tokyo, Japan

**Keywords:** colistin resistance, *mcr-1*, *Escherichia coli*, livestock, farmer, genotype, one health

## Abstract

Colistin is used to treat infectious diseases in humans and livestock; it has also been used as a feed additive for livestock for approximately 50 years. Since the *mcr-1* plasmid-mediated colistin resistance gene was discovered in China in 2015, it has been detected worldwide, mainly in livestock. In this study, we investigated the prevalence and characteristics of *mcr*-mediated colistin-resistant *Escherichia coli* in livestock and farmers in Japan. We collected fecal samples from 295 healthy livestock (202 cattle and 93 swine) and 62 healthy farmers from 72 livestock farms (58 cattle farms and 14 swine farms) between 2013 and 2015. Twenty-eight *mcr-1*-harboring *E. coli* strains were isolated from 25 livestock (six cattle and 19 swine) and three farmers (two cattle farmers and one swine farmer). The prevalence rates of *mcr-1*-harboring *E. coli* in livestock and farmers were 8.47 and 4.84%, respectively. Of the 28 strains, the resistance genes of three were transferable *via* the *mcr-1*-coding plasmids to *E. coli* J53 at low frequencies (10^−7^–10^−8^). Six strains coharbored *mcr-1* with CTX-M β-lactamases (CTX-M-14, CTX-M-27, or CTX-M-156). Of the isolates obtained from livestock and farmers in four farms (farms C, I, N, and P), nine strains had the same genotypical characteristics (sequence types and pulsed-field gel electrophoresis band patterns), plasmid characteristics (incompatibility group and plasmid transferability), and minimum inhibitory concentrations. Thus, the findings suggested that clonal strains could spread among livestock and farmers within farms. To our knowledge, this is the first study to detect clonal relatedness of *mcr-1*-mediated colistin-resistant *E. coli* in livestock and farmers. It is suggested that farmers are at a higher risk of acquiring *mcr-1*-harboring strains, calling for our attention based on the One Health concept.

## Introduction

Colistin is a cationic antimicrobial peptide that damages bacterial cell membranes by targeting the lipid A moiety of lipopolysaccharides (LPSs), which are present in the outer cell membrane of Gram-negative bacteria. Although the clinical usage of colistin induces strong adverse effects (i.e., renal dysfunction and neurotoxicity), it is still an extremely important antibiotic against infections caused by multidrug-resistant Gram-negative bacteria resistant to carbapenems and fluoroquinolones (Falagas and Kasiakou, 2005). In animals, colistin sulfate has been used as a therapeutic drug and feed additive worldwide. There is a concern that drug-resistant bacteria that have increased due to the use of antibiotics in livestock will not only make it difficult to treat infectious diseases in livestock but also make it difficult to treat infectious diseases in humans acquired through the consumption of livestock products. Therefore, the Food Safety Commission of Japan conducted a risk assessment of colistin sulfate for livestock in 2017 and estimated the risk posed by the drug as “Medium” (Food Safety Commission of Japan, 2017). In response to this estimation, the Japanese government shifted colistin sulfate for livestock as a therapeutic drug to a second-choice drug and withdrew colistin sulfate as a feed additive (Makita et al., 2020).

Colistin resistance is mediated by chromosomes or plasmids. The most common mechanism of chromosomally mediated colistin resistance is mainly through modification of the bacterial outer membrane through alteration of LPSs, by the addition of 4-amino-4-deoxy-l-arabinose and/or phosphoethanolamine, *via* amino acid changes in the PmrA/PmrB and PhoP/PhoQ two-component regulatory systems (Olaitan et al., 2014; Poirel et al., 2017; Aghapour et al., 2019). The other mechanism is the overexpression of efflux-pump systems or overproduction of capsule polysaccharide (Bengoechea and Skurnik, 2000; Campos et al., 2004). Plasmid-mediated colistin resistance is attributable to the transmissible gene *mcr-1*, which is a phosphoethanolamine transferase, leading to a more cationic LPS structure and consequently resistance to polymyxins (Liu et al., 2016). It was first discovered in swine in China in 2015; subsequently, *mcr-1*-harboring *Escherichia coli* has been reported worldwide, mainly in livestock and meat and even in humans (Nang et al., 2019). In addition, there are reports of the chromosomal localization of *mcr-1* in *E. coli* (Zurfluh et al., 2016; Li et al., 2018; Peng et al., 2019).

The spread of antimicrobial resistance genes should be controllable using the One Health concept, which involves the collaborative effort of health science professionals from multiple spheres toward attaining optimal health conditions for people, domestic animals, wildlife, plants, and the environment. The drivers of antimicrobial resistance include antimicrobial use and abuse in humans, animals, and the environment and the spread of resistant bacteria and resistance determinants within and between these spheres and around the world (McEwen and Collignon, 2018). Even in Japan, *mcr-1*-harboring *E. coli* strains have been isolated from livestock, imported and domestic meat, human clinical specimens, and the environment (Kusumoto et al., 2016; Nishino et al., 2017; Ohsaki et al., 2017; Tada et al., 2017; Hayashi et al., 2019). The possibility of crossing of the strains among the spheres is considered. Therefore, the relatedness of *mcr-1*-harboring strains from these different spheres needs to be determined based on the One Health concept.

The clonal relatedness of *mcr-1*-harboring *E. coli* from livestock on farms has been previously reported (Zheng et al., 2019). However, the clonal spread and relatedness of isolates of *mcr*-harboring *E. coli* from other spheres (e.g., among humans, livestock, and environment) remain unclear. To evaluate the relatedness of *mcr-1*-harboring *E. coli* isolated from livestock and farmers, we identified and compared the characteristics [sequence types (STs), pulsed-field gel electrophoresis (PFGE) band patterns, plasmid incompatibility (Inc) groups, plasmid transferability, and minimum inhibitory concentrations (MICs)] of the isolates. In this study, we investigated the prevalence, characteristics, and relatedness of *mcr*-mediated colistin-resistant *E. coli* in livestock (cattle and swine) and farmers in Japan.

## Materials and Methods

### Bacterial Isolates

Between 2013 and 2015, fecal samples from 295 healthy livestock (202 cattle and 93 swine) and 62 healthy livestock farmers (53 cattle farmers and nine swine farmers) were collected from 72 livestock farms (58 cattle farms and 14 swine farms) in the southern part of the Kyushu island, a major production area of cattle farming and swine farming in Japan. The livestock included 80 calves, 101 parent cattle, 21 fattening cattle (1–10 cattle per cattle farm), 26 piglets, 29 parent swine, and 38 fattening swine (3–10 swine per swine farm). Only one *E. coli* isolate was obtained per sample upon selection using Deoxycholate-hydrogen sulfide-lactose agar. The isolates were identified using matrix-assisted laser desorption ionization-time-of-flight mass spectrometry (MALDI-TOF MS; Vitek MS system; bioMérieux, Co., Ltd.). The studies involving human participants were reviewed and approved by the Ethical Review Committee at the Teikyo University School of Medicine (no.13–118). The participants provided written informed consent to participate in this study.

### Antimicrobial Susceptibility Testing

The antimicrobial susceptibilities to β-lactamase and levofloxacin (except for colistin) were determined using the agar dilution method (Clinical and Laboratory Standards Institute, 2020), and quality control was performed using *E. coli* ATCC 25922. The colistin concentration was determined using the broth dilution method. MICs were interpreted according to the breakpoints defined by the Clinical and Laboratory Standards Institute (CLSI) guidelines.

### Detection of Antimicrobial Resistance Genes

The presence of *mcr* genes, extended-spectrum beta-lactamase (ESBL)-encoding genes, and carbapenemase-encoding genes was determined using PCR and sequencing as described in previous studies (Dallenne et al., 2010; Poirel et al., 2011; Ye et al., 2016). DNA sequencing was conducted using the BigDye Terminator version 3.1 Cycle Sequencing kit (Applied Biosystems, Foster City, CA, United States) and an Applied Biosystems ABI3730xl analyzer (Thermo Fisher Scientific K.K.). BLAST version 1.12^1^ was used to process the sequencing data and identify the genes. Sequences were deposited in the GenBank database under accession numbers LC618530 through LC618535 for *bla*_CTX-M_ genes and LC618536 through LC618563 for *mcr-1* gene.

### Plasmid Incompatibility Groups and Conjugation Experiments

Plasmid Inc. groups were identified using PCR-based replicon typing (Carattoli et al., 2005; Johnson et al., 2012). Conjugation experiments were performed using a broth-mating method with *mcr-1*-harboring *E. coli* as the donor and sodium azide-resistant *E. coli* J53 as the recipient, as described previously (Nakano et al., 2004). Transconjugants were selected on Luria-Bertani agar plates containing colistin (4 mg/L) and sodium azide (100 mg/L).

### *Escherichia coli* Genotyping

For multilocus sequence typing (MLST), the protocol reported by Achtman et al. was applied to *E. coli* (Wirth et al., 2006). Seven housekeeping genes (*adk*, *fumC*, *gyrB*, *icd*, *mdh*, *purA*, and *recA*) were sequenced. DNA sequence variations were analyzed using the MLST database for *E. coli* ^2^ to determine STs. The similarity of the isolates was compared by PFGE of *Xba*I-digested genomic DNA (*Xba*I-PFGE) with a CHEF-MAPPER System (BioRad Laboratories, Japan), as previously described (Yano et al., 2013). PFGE was performed at 14°C for 20 h at 6 V/cm with a pulse time of 5.3–49.9 s, and the angle was set at 120°C. PFGE patterns were interpreted according to the criteria described by Tenover et al. (1995): “indistinguishable,” isolate with only one band different from that of the reference strain; “closely related,” isolate with 2–3 bands different from those of the reference strain; and “possibly related,” isolate with up to six bands different from those of the reference strain.

## Results

### Prevalence of *mcr-1*-Harboring *E. coli* Among the Livestock and Farmers

Colistin-resistant *E. coli* strains were isolated from 25 (8.47%) of the 295 livestock. The 25 strains were isolated from six cattle (three calves, two parent cattle, and one fattening cattle) from five farms and 19 swine (15 piglets, one parent swine, and three fattening swine) from 11 farms. All strains harbored *mcr-1*, as confirmed by PCR and sequencing. The prevalence of *mcr-1*-harboring *E. coli* strains was 2.97% (6/202 strains) for cattle and 20.43% (19/93 strains) for swine. *mcr-1*-harboring *E. coli* strains were also isolated from three healthy farmers (two cattle farmers and one swine farmer; 4.84%) among 62 farmers. The prevalence of *mcr-1*-harboring *E. coli* strains was 3.77% (2/53 strains) for cattle farmers and 11.11% (1/9 strains) for swine farmers.

### Antimicrobial Susceptibility and Resistance Genes

The antimicrobial susceptibilities of 28 *mcr-1*-harboring *E. coli* strains are shown in Table 1. All the strains were resistant to colistin (4–16 mg/L), and 16 strains were co-resistant to levofloxacin (≥8 mg/L). Carbapenem-resistant strains were not found, and six strains were resistant to cefpodoxime (≥4 mg/L) and coharbored the CTX-M β-lactamase gene. The *bla*_CTX-M_ genotype belongs to the CTX-M-9 group (three CTX-M-27 strains and one CTX-M-14 strain) and CTX-M-1 group (two CTX-M-156 strains).

**Table 1:** Characteristics of *mcr-1*-harboring *Escherichia coli* isolated from livestock and farmers.

Farm	Strains	Source	Resistance gene	Sequence type	PFGE pattern	Plasmid incompatibility group^a^	Conjugation efficiency^b^	MIC (mg/L)^c^			
								CPDX	IPM	LVFX	CL
A	TK3004	piglet	CTX-M-14	744	-	F	No	128	0.125	16	8
B	TK3067	piglet	-	165	-	F, X4	No	0.5	0.125	16	8
C	TK3085	piglet	-	744	A	F, FIB, X1, X4	No	0.5	0.125	32	8
	TK3091	fattening swine	-	744	A	F, FIB, X1, X4	No	1	0.125	32	8
D	TK3105	fattening cattle	-	95	-	F, FIB, FIC, I1Iγ	No	1	0.25	0.125	8
E	TK3121	piglet	-	10	-	F, FIB, HI1, I1Iγ	No	0.5	≤0.06	≤0.06	8
	TK3125	parent swine	-	34	-	F, I1Iγ, Y	No	1	0.25	0.125	8
F	TK3140	piglet	-	398	-	X1, Y	No	1	0.125	4	8
G	TK3149	piglet	-	48	-	F, FIB, HI2	No	0.5	0.125	1	8
H	TK3157	piglet	-	349	-	HI2, P, X1	No	2	0.125	4	8
I	TK3166	piglet 1	-	5229	-	F, FIB	No	1	0.25	32	8
	TK3965	piglet 2	-	746	B	F, FIB, A/C	No	4	0.25	32	8
	TK3981	swine farmer	-	746	B	F, FIB, A/C	No	4	0.125	32	8
J	TK3226	calf	CTX-M-156	617	-	F	No	256	0.125	16	4
K	TK3238	calf	-	69	-	HI1, I1Iγ	No	0.5	0.125	≤0.06	8
L	TK3247	piglet	-	10	-	F, FIB, HI2, I1Iγ	No	0.5	≤0.06	1	8
M	TK3347	parent cattle	-	106	-	F, FIA, FIB	No	0.5	0.125	≤0.06	8
N	TK3469	piglet 1	-	93	-	F, FIB, I1Iγ, X4, Y	No	0.5	0.125	8	8
	TK3479	piglet 2	-	88	-	F, FIB, Y	No	0.5	0.125	0.5	4
	TK3473	piglet 3	-	746	C	F, FIB, X4	No	2	0.125	32	8
	TK3491	piglet 4	-	746	C	F, FIB, X4	No	4	0.25	32	8
	TK3500	piglet 5	-	10	-	F, FIB, HI2, I1Iγ	No	1	≤0.06	8	16
O	TK3562	fattening swine 1	-	Novel^d^	-	F, FIB, FIC	No	1	0.125	≤0.06	4
	TK3573	fattening swine 2	-	410	-	F, FIB, I1Iγ	No	1	0.125	32	8
P	TK5269	calf	CTX-M-27	2929	D’	NT	1.0 × 10^−7^	>256	0.125	16	8
	TK5276	parent cattle	CTX-M-27	2929	D	NT	4.2 × 10^−8^	256	0.125	8	8
	TK5282	cattle farmer	CTX-M-27	2929	D	NT	1.3 × 10^−7^	>256	0.125	8	4
Q	TK5359	cattle farmer	CTX-M-156	10	-	F, P	No	256	0.125	4	8

a NT, nontypeable.

b No, not transferable.

c CPDX, cefpodoxime; IPM, imipenem; LVFX, levofloxacin; CL, colistin.

d Novel type consisting of the following allelic combination: *adk 6*, *fumC 474*, *gyrB 32*, *icd 109*, *mdh 8*, *purA 1*, and *recA 2*.

### Genetic Characteristics of *mcr-1*-Harboring *E. coli*

All *mcr-1*-harboring *E. coli* strains were genotyped using MLST analysis (Table 1). Six *mcr-1*-harboring *E. coli* strains from cattle belonged to five different STs (two strains belonged to ST2929 and one strain each to ST69, ST95, ST106, and ST617). Nineteen *mcr-1*-harboring *E. coli* strains from swine belonged to 13 different STs (three to ST10, three to ST744, and three to ST746, followed by 10 STs). The STs of *E. coli* isolated from cattle completely differed from those of *E. coli* isolated from swine. *mcr-1*-harboring *E. coli* strains from three farmers belonged to three different STs (ST10, ST746, and ST2929). All *mcr-1*-harboring *E. coli* strains possessed diverse types of plasmid Inc. Of the 28 strains, 22 possessed F replicon regions, 17 possessed IncFIB, eight possessed IncI1Iγ, six possessed IncX4, four possessed IncHI2, four possessed IncX1, four possessed IncY, three possessed nontypeable, two possessed IncA/C, two possessed IncFIC, two possessed IncHI1, and two possessed IncP. By performing conjugation experiments, transconjugants containing the *mcr-1*-encoding plasmid were obtained at low frequencies (1.0 × 10^−7^ to 4.2 × 10^−8^) from three strains. These three strains coharbored the CTX-M-27 β-lactamase gene with a nontypeable Inc. plasmids and were isolated from farm P.

### Clonal Relatedness of *mcr-1*-Harboring *E. coli* Among Livestock and Farmers

Nine strains from four farms were found to have similar CTX-M types, STs, Inc. group, plasmid transferability, and MICs. The strains were as follows: TK3085 and TK3091 from farm C; TK3965 and TK3981 from farm I; TK3473 and TK3491 from farm N; and TK5269, TK5276, and TK5282 from farm P. *Xba*I-PFGE was used to confirm the clonal relatedness of the strains among the farms (Table 1). Five PFGE band patterns were obtained for the nine strains. Each PFGE pattern represented only one farm: Pattern A was noted in the farm C strains; pattern B in the farm I strains; pattern C in the farm N strains; and patterns D and D’ in the farm P strains. The PFGE D’ band patterns differed by three bands from the D band patterns; the strain with the D’ band patterns may have been derived from that with the D band patterns. The strains with similar PFGE band patterns were considered to represent clonal strains.

## Discussion

*mcr-1*-harboring *E. coli* has increased worldwide. The prevalence of *mcr-1*-harboring *E. coli* in healthy livestock has been reported to be as follows: 0.6% in Great Britain in 2013–2015 (from swine); 4.45% in Thailand (from swine); 71.43, 68.86, and 87.58% in China in 2015–2016 (from cattle, swine, and chickens, respectively; Duggett et al., 2017; Zhang et al., 2019; Khine et al., 2020); and 0.42% in Japan in 2000–2014 (cattle, swine, and chickens; Kawanishi et al., 2017). Our study revealed a high prevalence of *mcr-1*-harboring *E. coli* in healthy livestock (8.47%); the prevalence in swine (20.43%) was higher than that in cattle (2.97%). Furthermore, we found that the prevalence was 4.84% in farmers in Japan. A previous study showed that the prevalence rates of *mcr-1*-harboring *E. coli* strains were 1.43 and 0.65% in inpatients, wherein it was associated with infection, and healthy volunteers, respectively, in China in 2007–2015 (Wang et al., 2017); our study showed comparatively higher distribution among livestock farmers.

The high prevalence is likely to be associated with colistin use (Tong et al., 2018). Continuous selection pressure might have promoted the dissemination of *mcr-1*, ultimately resulting in its exceedingly high prevalence in livestock. Colistin is no longer used as a feed additive in some countries, such as the United States of America and the EU (European Commission, 2001; European Medicines Agency, 2016). Similarly, the use of colistin as a feed additive in livestock farms was banned from July 2018 in Japan; 52.5% of test farms showed a decrease in the prevalence of *E. coli* with plasmid-mediated colistin resistance 12 months after colistin use was banned in Japan (Makita et al., 2020).

We determined the genetic characteristics of 28 *mcr-1*-harboring *E. coli* strains isolated from livestock and farmers. The genotypes of the 28 strains consisted of 18 different STs; dominant STs were not observed. Major genotypes of ESBL-producing *E. coli* in humans, such as ST131 (Nicolas-Chanoine et al., 2014), were not detected in this study. Seven strains (four strains, ST10; one strain, ST34; one strain, ST48; and one strain, ST617) were found to belong to clonal complex 10 (CC10), including two strains of CTX-M-156 coharboring *E. coli* CC10 (ST10 and ST617), which is considered to have a broad range of hosts, including humans, animals, vegetable, wastewater, and urban streams (Manges et al., 2015; Varela et al., 2015; Reid et al., 2019; Massella et al., 2020). Furthermore, the strains harboring antimicrobial resistance genes were frequently coharboring virulence-associated genes. There is growing concern about the spread of *E. coli* CC10 across the spheres.

Three *mcr-1*-harboring *E. coli* strains (TK5269, TK5276, and TK5282) isolated from a calf, parent cattle, and cattle farmer in farm P had transferable resistance genes to *E. coli*. J53, which is considered as a plasmid-mediated colistin-resistant *E. coli*. We also isolated *mcr-1*-harboring *Klebsiella pneumonia* from the same individual (calf of farm P; data not shown). Herein, we selected only one *E. coli* from one sample, even though there was a possibility that *mcr-1*-harboring *E. coli* strains with different characteristic (STs, Inc. groups, or PFGE band patterns) were present in these samples. There is also a possibility of *mcr-1*-coding gene transfer across different species and strains by horizontal gene transfer, such as conjugation and transformation. A recent study reported that *E. coli* strains harboring chromosomally encoded *mcr-1* were isolated from 3.5% of *mcr-1*-positive *E. coli* in China from 2016 to 2018 (Shen et al., 2020). Therefore, the whole genome of the other 25 strains of *mcr-1*-positive *E. coli* should be analyzed to characterize the genetic construction and determine whether the *mcr-1* gene is encoded on a plasmid or chromosome in the future.

Since resistance genes (e.g., *mcr-1*, ESBL-encoding genes, and carbapenemase-encoding genes) are being detected in humans, animals, and the environment worldwide, it is necessary to take comprehensive measures to prevent the spread based on the One Health concept (Baquero et al., 2019). It is considered that one of the epidemic routes of resistant bacteria is from livestock to humans, but ESBL-harboring strains from livestock and humans show low relatedness (Dorado-García et al., 2018; Mughini-Gras et al., 2019). On the contrary, ESBL-harboring strains from companion animals and humans show high relatedness (Hong et al., 2019). Our study revealed the clonal relatedness of *mcr-1*-harboring *E. coli* isolated from livestock and farmers. It is suggested that farmers are at a higher risk than individuals living outside farms because farmers come into close contact with livestock every day, such as companion animals.

In conclusion, we determined the prevalence of *mcr-1*-harboring *E. coli* in healthy livestock (8.47%) and farmers (4.84%). Some strains with the same genotype characteristics (ST and PFGE band patterns), plasmid characteristics (Inc groups and plasmid transferability), and MICs were isolated from livestock and farmers at the farms. Thus, the findings suggest the spread of *mcr-1*-mediated colistin-resistant *E. coli* among livestock and farmers within the farms studied. To our knowledge, this is the first study to identify the clonal relatedness of *mcr-1*-mediated colistin-resistant *E. coli* in livestock and farmers. Based on the concept of One Health, it is required to identify the association of *mcr-1*-harboring *E. coli* among healthy individuals, hospital patients, animals, wastewater of the livestock, and river water by characterizing the isolates.

## Data Availability Statement

The data presented in the study are deposited in the GeneBank database repository, accession numbers (LC618530 through LC618563).

## Ethics Statement

The studies involving human participants were reviewed and approved by Ethical Review Committee at the Teikyo University School of Medicine (no.13–118). The patients/participants provided their written informed consent to participate in this study.

## Author Contributions

AN and RNa: conceptualization, methodology, investigation, and writing – original draft. RNi: conceptualization and resources. YS, SH, TK-U, and TU: validation and data curation. YO and HY: supervision and project administration. All authors contributed to the article and approved the submitted version.

### Conflict of Interest

The authors declare that the research was conducted in the absence of any commercial or financial relationships that could be construed as a potential conflict of interest.

## Footnotes

^1^https://blast.ncbi.nlm.nih.gov/Blast.cgi

^2^https://pubmlst.org/bigsdb?db=pubmlst_mlst_seqdef
